# The Site of Lymph Node Metastasis: A Significant Prognostic Factor in Pancreatic Ductal Adenocarcinoma

**DOI:** 10.5146/tjpath.2022.01583

**Published:** 2022-09-15

**Authors:** Anil Aysal, Cihan Agalar, Sumru Cagaptay, Turugsan Safak, Tufan Egelı, Mucahit Ozbılgın, Tugba Unek, Tarkan Unek, Ozgul Sagol

**Affiliations:** Department of Pathology, Dokuz Eylul University, Faculty of Medicine, Izmir, Turkey; Department of General Surgery, Dokuz Eylul University, Faculty of Medicine, Izmir, Turkey; Department of Medical Oncology, Dokuz Eylul University, Faculty of Medicine, Izmir, Turkey

**Keywords:** Pancreatic ductal adenocarcinoma, Regional lymph node site, Metastasis

## Abstract

***Objective:*** While the presence and number of metastatic lymph nodes (LNs) are important prognostic factors for pancreatic ductal adenocarcinoma (PDAC), there is no recommendation to specify metastatic regional LN localization in the current staging system. The aim of this study was to evaluate the prognostic effect of regional metastatic LN localizations in PDAC.

***Material and Method:*** Metastatic sites of 101 consecutive PDAC patients who underwent pancreaticoduodenectomy were classified as peripancreatic, perigastric, hepatica communis, hepatoduodenal, and superior mesenteric artery. The frequency of metastasis in each region and the association between the presence of metastasis in each site and overall and disease-free survival were statistically analyzed.

***Results:*** Eighty cases (79.2%) had peripancreatic, 7 (6.9%) had perigastric, 6 (5.9%) had hepatica communis, 7 (6.9%) had hepatoduodenal, and 4 (4%) had superior mesenteric artery LN metastasis. The overall and disease-free survival values were significantly shorter in patients with hepatoduodenal LN metastasis (log rank; p= 0.001, p=0.017, respectively). The presence of metastatic superior mesenteric artery LN was significantly associated with shorter disease-free survival in univariate analysis (p=0.017). Hepatoduodenal LN metastasis was an independent predictor of mortality (p=0.005) in multivariate analysis.

***Conclusion:*** The presence of hepatoduodenal LN metastasis is an independent poor prognostic factor for mortality. The presence of metastatic LN in the superior mesenteric artery region was significantly associated with shorter disease-free survival time, although not an independent predictor. We conclude that the metastatic regional LN sites, especially the hepatoduodenal region, have an impact on the prognosis, and should be included in synoptic pathology reports.

## INTRODUCTION

Pancreatic ductal adenocarcinomas (PDACs) are aggressive tumors with a 5-year survival rate around 10%. The most important prognostic parameters for these tumors include tumor size, pathological stage, presence of lymph node metastasis, number of metastatic lymph nodes, and vascular invasion. Regarding regional lymph node metastases, it is recommended to specify the number of metastatic lymph nodes and pN stage, while metastatic regional lymph node localization does not have a place in the current staging system (1). According to the 2014 consensus of the International Pancreatic Surgery Working Group, standard lymphadenectomy for pancreatoduodenectomy covers the lymph node regions of 5, 6, 8a, 12b1, 12b2, 12c, 13a, 13b, 14a, 14b, 17a, and 17b, while for distal pancreatectomy, the dissection of lymph node regions of 10, 11, and 18 is considered as standard (2). While regional lymphatic metastases are initially expected to occur in primary lymph nodes (pancreaticoduodenal - regions 13 and 17), second line lymph nodes (inferior pyloric, hepatica communis, and hepatic pedicle/hepatoduodenal – regions 6, 8, and 12) are involved as the disease progresses (3). There are few studies on the relationship between the presence of metastases in non-pancreaticoduodenal regional lymph nodes and the prognosis that demonstrate the association between various metastatic lymph node localizations and the prognosis (3–10). As the data on this issue are very limited, the aim of this study was to evaluate the effect of regional metastatic lymph node localization on the prognosis of PDAC.

## MATERIAL and METHODS

The study protocol was approved by the institutional ethics committee (Approval date and number: 2022/18-15). One hundred and one patients who underwent pancreaticoduodenectomy (Whipple procedure) between 2010 and 2020 with no history of neoadjuvant chemotherapy and were diagnosed as PDAC at our institution were included. The cases that died within the first 3 months of the surgery (i.e., perioperative period) were excluded. Patient demographics, perioperative mortality, and oncological follow-up info were retrieved from the patient records. Survival status and dates of death were obtained from the national population registry system and the hospital registry system. The histopathologic features including tumor differentiation, tumor size, pT, pN, metastatic lymph node number, lymphovascular invasion, and perineural invasion were obtained from pathology reports. Standard lymphadenectomy protocols had been performed for all cases (2). The regional lymph node localizations were classified as peripancreatic and perigastric lymph nodes (dissected by the pathologist (A.A., S.C. and O.S.) from the main specimen), and hepatica communis, hepatoduodenal/hepatic pedicle and superior mesenteric artery lymph nodes (separately resected and specified by the surgeons during the operation). Lymph node dissection had been performed from all these regions in each case in the study. Peripancreatic lymph nodes had been sampled without further categorization. Each lymph node had been completely sampled and examined. Hematoxylin-eosin stained slides were examined for the presence and number of metastatic lymph nodes in each region. The associations between the presence of metastasis in each metastatic lymph node site and the histopathologic prognostic factors and the overall and disease-free survival were determined statistically.

Statistical analyses were performed using the SPSS 24.0 statistical package (SPSS, Chicago, IL). Categorical data were compared using the Chi-square test and Fisher Exact test. The independent samples T-test was used for comparing normally distributed continuous variables, and the non-normally distributed variables were compared using the Mann–Whitney U test. Overall survival (OS) was determined as the duration between the operation date and time of death or last follow-up, while the duration between the operation date and time of recurrence was calculated to determine disease-free survival (DFS). The Kaplan-Meier (K-M) estimator was used to calculate the OS and DFS rates, and the Log-rank test was used to compare the differences between survival curves. The association between OS and DFS, and the prognostic parameters were also analyzed by multivariate analysis. Log and Cox regression tests were used to analyze the association between the survival time and potential predictors. A p value<0.05 was considered statistically significant.

## RESULTS

### Clinicopathologic Features

The mean age was 63.7 ± 10.44 (range: 37-89) years. Sixty-two patients (61.3%) were male with a male:female ratio of 1.58. The tumor was well differentiated in 68 (67.3%), moderately differentiated in 26 (25.7%), and poorly differentiated in 7 patients (7%). The surgical margin was positive in 60 cases (59.4%), and the retroperitoneal surgical margin was most commonly involved (n=43). Lymphovascular invasion was present in 78 cases (77.2%), whereas perineural invasion was detected in 87 (86.1%). pT stage was pT1 in 7, pT2 in 25, and pT3 in 68 patients, and pT4 in one patient. The mean number of dissected lymph nodes was 24.8 ± 10.38 (range: 4-56), and the mean number of metastatic lymph nodes was 3.08 ± 3.00 (range: 0-14). Nineteen patients did not have lymph node metastasis, while 62 were pN1 and 20 were pN2. Almost all of the cases (97.5%) with lymph node metastasis had peripancreatic lymph node metastasis.

**Figure 1:**
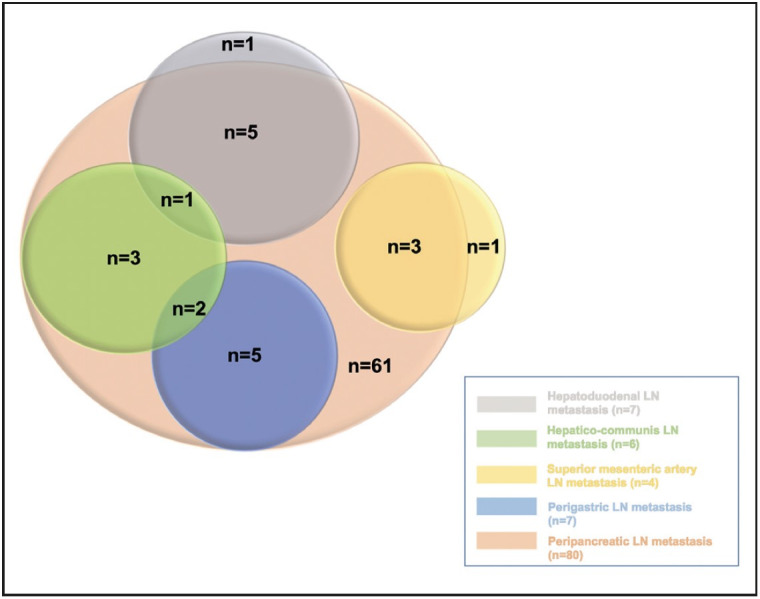
Venn diagram showing the relationship between all metastatic lymph node regions (with the number of cases with metastasis in that region).

Eighty cases (79.2%) had peripancreatic, 7 cases (6.9%) had perigastric, 6 cases (5.9%) had hepatica communis, 7 cases (6.9%) had hepatoduodenal/hepatic pedicle, and 4 cases (4%) superior mesenteric artery lymph node metastasis (Table 1). All but one of the cases with hepatoduodenal lymph node metastasis also had peripancreatic lymph node metastasis. Almost all cases with extra-peripancreatic lymph node metastases (except 2 cases) were already accompanied by peripancreatic LN metastasis. The relationship between all metastatic lymph node regions is shown in Figure 1 (together with the number of cases with metastasis in that region). Extended lymphadenectomy had been performed in six patients.

**Table 1:** The frequencies of metastatic regional lymph node sites and the results of univariate analysis for overall and disease-free survival.

**Regional lymph node site**	**Cases** **n(%)**	**Log rank** **(OS)**	**Log rank** **(DFS)**
**Pancreatoduodenal (Ln13+17)** Metastatic Nonmetastatic	80 (79.2) 21 (20.8)	**p=0.014**	p=0.773
**Perigastric (Ln5+6)** Metastatic Nonmetastatic	7 (6.9) 94 (93.1)	p=0.811	p=0.411
**Hepatoduodenal/hepatic pedicle (Ln12)** Metastatic Nonmetastatic	7 (6.9) 94 (93.1)	** ** **p=0.001**	** ** **p=0.017**
**Hepatica communis (Ln8)** Metastatic Nonmetastatic	6 (5.9) 95 (94.1)	p=0.325	p=0.113
**Superior mesenteric (Ln14)** Metastatic Nonmetastatic	4 (4) 97 (96)	p=0.91	**p=0.017**

**OS:** Overall survival, **DFS:** Disease-free survival. The parameters significant in univariate analysis are bold.

Positive surgical margin status was statistically associated with the presence of peripancreatic lymph node metastasis (68.3% vs. 86.7%, p=0.025), and hepatoduodenal lymph node metastasis (0% vs. 11.7%, p=0.039). The presence of lymphovascular invasion showed a statistically significant association only with the presence of peripancreatic lymph node metastasis (39.1% vs. 91%, p<0.001). The site of the metastatic lymph node was not associated with other histopathologic prognostic factors such as tumor differentiation, pT, and perineural invasion.

### Results of the Survival Analysis

The mean follow-up time was 21.3 ± 26.5 months. Seventy-one (70.3%) cases had recurrence, while 84 patients (83.2%) died during follow-up. The average OS was 27.5 months (± 26.26). The average DFS was 9.6 months (± 9.9). The OS rates at 1, 3, 5 years were 68.7%, 31%, and 15%, respectively. The DFS rate at 1 year was 26.8%.

The mortality and recurrence rates were not statistically related to any of the metastatic lymph node regions.

In survival analysis, the estimated OS was significantly shorter in patients with pN1 and pN2 stage compared to patients without lymph node metastasis (56.42 vs. 31.36 vs. 16.40 months, log rank; p=0.002), and also with moderate and poor tumor differentiation (40.24 vs. 23.84 vs. 8.79 months, log rank; p<0.001), positive surgical margin (46.05 vs. 26.37 months, log rank; p=0.008), lymphovascular invasion (54.43 vs. 27.64 months, log rank; p=0.003), and perineural invasion (55.88 vs. 29.48 months, log rank; p=0.011). The DFS was also significantly shorter in patients with moderate and poor tumor differentiation (19.27 vs. 13.52 vs. 5.66 months, log rank; p=0.025).

**Figure 2:**
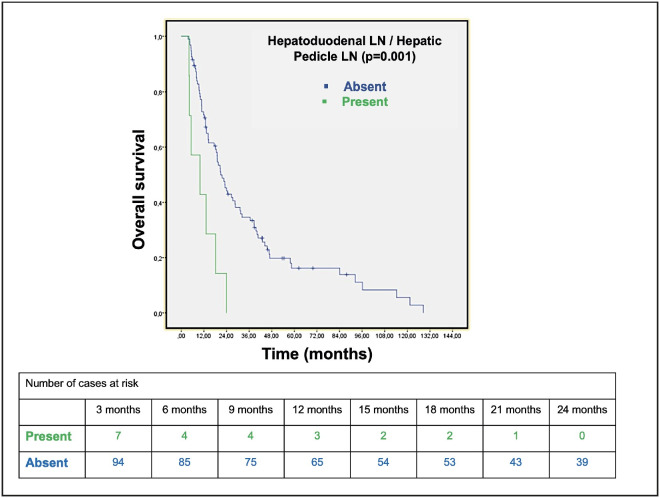
Kaplan-Meier overall survival curve of the hepatoduodenal/hepatic pedicle lymph node metastasis, with the risk table showing the number of the cases at risk.

**Figure 3:**
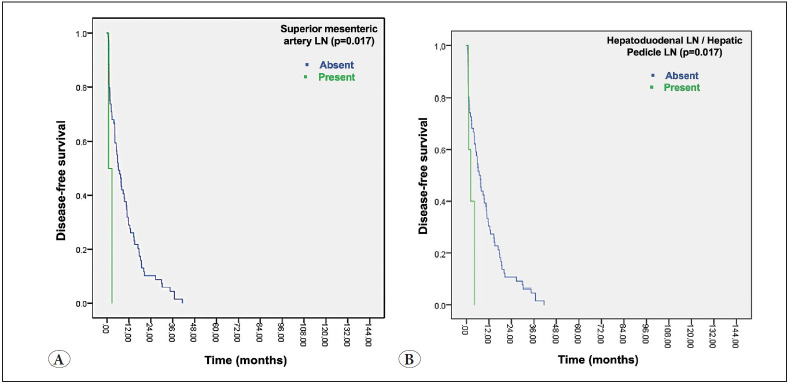
Kaplan-Meier survival curves of the groups. **A)** Disease-free survival for superior mesenteric artery lymph node metastasis, **B)** Disease-free survival for hepatoduodenal lymph node metastasis.

The OS (35.5 vs. 11.24 months) and DFS (10.17 vs. 2.6 months) were significantly shorter in patients with hepatoduodenal/hepatic pedicle lymph node metastasis (log rank; p=0.001, p=0.017, respectively). The OS (35.5 vs. 11.24 months) and DFS (10.17 vs. 2.6 months) were significantly shorter in patients with hepatoduodenal/hepatic pedicle lymph node metastasis (log rank; p=0.001, p=0.017, respectively) (Figure 2). The DFS was significantly shorter in patients with superior mesenteric artery lymph node metastasis (9.8 vs. 1.8 months) (log rank; p=0.017) (Figure 3). The OS was shorter in patients with hepatica communis lymph node metastasis as well, albeit not statistically significant (34.7 months vs. 20.5 months; p=0.32) (Table 1).

In multivariate analysis, hepatoduodenal/hepatic pedicle lymph node metastasis was found to be an independent predictor of mortality with the Cox regression analysis (HR 3.27, 95% CI: 1.43 to 7.46, *p*=0.005) (Table 2). No independent predictors of recurrence were detected with the Cox regression analysis.

**Table 2:** Multivariate analysis of predictors of overall survival.

**Parameters**	**p**	**HR (95%CI)**
**Tumor differentiation**	**<0.001**	5.88 (2.46-14.03)
Lymphovascular invasion	0.537	1.31 (0.55-3.1)
Perineural invasion	0.293	1.68 (0.63-4.45)
**Hepatoduodenal LN metastasis**	**0.005**	3.27 (1.43-7.46)
Peripancreatic LN metastasis	0.183	1.61 (0.79-3.28)
Positive surgical margin	0.295	1.31 (0.78-2.18)

**HR:** hazard ratio. The parameters significant at the multivariate analysis are bold.

## DISCUSSION

Lymph node metastasis, specifically when present in the hepatoduodenal/hepatic pedicle region, was found to be an independent indicator of mortality in this study. Although not an independent indicator, the DFS was significantly shorter in patients with superior mesenteric artery lymph node metastasis. The presence of hepatica communis lymph node metastasis was also found to be associated with a shorter life expectancy of 14 months, which is a very important period for these patients due to the low life expectancy of PDAC, albeit without statistical significance.

All efforts for many years to improve the prognosis of pancreatic ductal adenocarcinoma have yet to yield satisfactory results. Late diagnosis, lack of effective treatment options, and inadequate prediction of the prognosis, which can be considered the main problems related to this tumor, have been topics that have been studied to date. A significant change that has occurred in recent years as a result of studies on PDAC for better prognostic classification is the transformation of PDAC pathological staging into a tumor size-based staging system, which is a more objective assessment, rather than tumor spread-based staging, which was more prone to erroneous assessments (1,11). In addition, lymph node substaging has been added to pN staging due to its significant prognostic contribution (1,12). On the other hand, the use of neoadjuvant therapy and, albeit limited, targeted therapy options are among the leading developments in treatment (13,14). Despite all these developments, the 5-year survival is still reported as 11% according to current data (15). Our study showed a slightly higher 5-year survival rate (15%), with an average survival time of 27.5 months.

Numerous studies have shown that the assessment of the number of metastatic lymph nodes contributes much more to the prognostic classification than the previous assessment based on the presence/absence of metastases alone (12,16–18). The utility of the lymph node ratio in predicting prognosis has also been emphasized in the literature, although it has not yet been accepted as a definitive prognostic determinant (17–19).

Regional lymph node metastases mostly occur in pancreatoduodenal lymph nodes, with a high percentage (4–6) and extended lymphadenectomy does not improve prognosis (2), as detected in our study. While the prognostic effects of the number and the ratio of regional lymph node metastasis have been highly emphasized, the studies investigating the effect of metastatic lymph node sites especially in non-pancreatoduodenal regions on prognosis are very limited and reveal conflicting data (Table 3) (3–10).

**Table 3:** The summary of the findings in the studies investigating the effect of non-pancreatoduodenal metastatic lymph node sites on prognosis in cases with PDAC who underwent pancreatoduodenectomy.

**Study**	**Case number**	**Case population**	**Regional lymph node site evaluated**	**Detection rate (%)**	**The effect on prognosis**
Wennerblom et al. (3)	66	Pancreatic and periampullary adenocarcinoma	Hepatica communis (Ln8)	21	Negative prognostic
Cordera et al. (7)	55	PDAC	Hepatica communis (Ln8)	18	Negative prognostic
LaFemina et al. (8)	147	PDAC	Hepatica communis (Ln8)	16	Negative prognostic
Philips et al. (9)	247	PDAC	Hepatica communis (Ln8)	20.8	No prognostic effect
Malleo et al. (5)	255	PDAC	-Superior mesenteric artery (Ln14) -Hepatoduodenal (Ln12) -Hepatica communis (Ln8)	55.2 34.1 16.2	Negative prognostic No prognostic effect No prognostic effect
Malleo et al. (6)	424	PDAC	-Superior mesenteric artery (Ln14) -Hepatoduodenal (Ln12) -Hepatica communis (Ln8) -Jejunal mesentery	57.5 20.5 15.9 9.9	Negative prognostic No prognostic effect Impact not discussed Negative prognostic
Golse et al. (10)	100	PDAC and ampullary carcinoma	-Superior mesenteric artery (Ln14) -Hepatoduodenal (Ln12) -Hepatica communis (Ln8)	5.4 0.9 6.4	Negative prognostic No prognostic effect Negative prognostic
Imamura et al. (4)	360	PDAC	-Superior mesenteric artery (Ln14) -Hepatoduodenal (Ln12) -Hepatica communis (Ln8) and celiac (Ln9) -Jejunal mesentery	26.7 12.7 11.8 11.1	No prognostic effect Negative prognostic No prognostic effect No prognostic effect

**PDAC:** Pancreatic ductal adenocarcinoma.

Wennerblom et al. have detected hepatica communis lymph node metastasis in 21% of the cases that underwent pancreatoduodenectomy and showed that the presence of hepatica communis lymph node metastasis was associated with significantly shorter survival in contrast to the patients with no metastasis in this region (3). However, the study group included periampullary adenocarcinomas as well as pancreatic carcinomas, which caused heterogeneity in terms of the case population (3). In two different studies of pure pancreatic ductal adenocarcinoma cases that underwent pancreatoduodenectomy, the negative prognostic effect of the presence of hepatica communis lymph node metastasis (with 18% and 16% detection rates) on survival were demonstrated statistically (7,8). On the other hand, two other studies showed that metastases in this region did not have an effect on prognosis and survival, revealing contrasting data on the subject (6,9). The common feature of these studies is that, unlike our study, they evaluated the prognostic effect of metastases only in the hepatica communis lymph node region among the regional lymph node sites. In our study, in which the prognostic value of all regional lymph node localizations were separately evaluated, hepatica communis lymph node metastasis was interestingly detected at a lower rate (5.9%) than in these studies. However, we found that the presence of hepatica communis lymph node metastases was associated with a shorter life expectancy of 14 months, in support of most of these studies, although there was no statistical significance probably due to the low detection rate.

In another study with a design similar to ours, Malleo et al. investigated the prognostic effects of the presence of hepatica communis, superior mesenteric artery, and hepatoduodenal lymph node metastases, and found that only the presence of superior mesenteric artery metastasis was an independent prognostic factor in predicting shorter survival time (5). The authors have confirmed their findings in another very recent study with a much larger number of cases and prospective design, and have also added the finding that jejunal mesenteric lymph node metastasis was similarly associated with the prognosis (6). In our study, which had a lower number of cases and where the jejunal mesentery lymph nodes were not separately sampled, the rate of superior mesenteric artery lymph node metastasis was found to be quite low, and metastasis at this site was significantly related to shorter disease-free survival time, although not an independent predictor. Such a low rate is probably due to populational differences. In a study from France, with detection rates of SMA and hepatica communis lymph node metastases were similar to ours and a case number was close to ours despite a more heterogeneous patient population (composed of PDAC and ampullary carcinoma), Golse et al. showed that superior mesenteric artery and hepatica communis lymph node metastasis were significantly associated with shorter overall survival in univariate analysis, although the significance was lost in multivariate analysis (10). This, together with our findings, may indicate that the rate of metastasis in these regions may be lower in the European population. In addition, when they evaluated only the cases with PDAC, they found that hepatica communis lymph node metastasis was an independent prognostic factor for disease-free survival.

In a study also including patients who underwent distal pancreatectomy, where only pancreatic head tumors were examined, it was shown that only hepatoduodenal lymph node metastasis among regional lymph node localizations was associated with a very poor prognosis, with a 5-year survival rate of 0% especially in uncinate process tumors, although multivariate analysis was not performed (4). They also stated that the prognostic value of jejunal mesenteric lymph node metastasis is low. To the best of our knowledge, that study is the first to indicate the negative prognostic effect of the presence of hepatoduodenal lymph node metastasis (4), and our study is the second on this subject but the first to demonstrate that hepatoduodenal lymph node metastasis is an independent prognostic predictor for overall survival, providing stronger evidence. No significance was detected in the few other studies that evaluated the prognostic effect of hepatoduodenal lymph node metastasis (5,6,10).

Twelve is an important number specified as the minimum number of lymph nodes to be examined for pancreatoduodenectomy specimens for optimal staging (20). The mean number of evaluated lymph nodes was 24 in our study, with a maximum number of 56, and more than 30 lymph nodes were evaluated in almost one third of the patients. The reasons for the high number of lymph nodes evaluated are the total sampling of peripancreatic adipose tissue of the main specimen in our pathology department, and the fact that all regional lymph node stations are dissected separately and meticulously with a standard approach in each case by our surgeons. This approach may have a slight positive effect on our survival rates; however, we think that the high number of lymph nodes evaluated in our series and the complete sampling of each lymph node are the features that increase the reliability of the data we present here.

The major limitations of this study were its retrospective design and the relatively low number of cases compared to most studies evaluating the prognostic effects of various regional lymph node sites. The peripancreatic lymph nodes were not subcategorized due to the retrospective nature of our study, and this may be considered as another limitation. Nevertheless, we think that strong evidence that can shed light on the topic has been obtained in this study as we included a relatively homogeneous patient population (including only the PDAC patients that had undergone pancreatoduodenectomy without neoadjuvant therapy) with the majority having a high lymph node count.

It is clear that there is confusion in the literature in terms of the prognostic value of metastatic regional lymph node localizations (Table 3). On the other hand, it seems undeniable that the presence of metastases in the hepatica communis, superior mesenteric artery, and hepatoduodenal lymph node regions has a negative effect on the prognosis, according to the few studies providing strong evidence. In our study, all but one of the cases with hepatoduodenal lymph node metastasis also had peripancreatic lymph node metastasis. Moreover, in multivariate analysis, the presence of peripancreatic lymph node metastasis was not statistically significant, while hepatoduodenal lymph node metastasis was found to be a significant and an independent prognostic factor. For these reasons, it can be assumed that the prognostic effect of hepatoduodenal lymph node metastasis is independent of the effect of peripancreatic lymph node metastasis. Our finding and strong opinion regarding hepatoduodenal lymph node metastasis is based on these factors, although it was detected only in 7 cases.

In conclusion, this is the first study to show the presence of hepatoduodenal/hepatic pedicle lymph node metastasis as an independent poor prognostic factor for mortality risk in patients with PDAC, although it was detected in a low number of cases. The presence of lymph node metastasis in the superior mesenteric artery region is significantly associated with shorter disease-free survival time, although not an independent predictor. We conclude that the metastatic lymph node site has an impact on the prognosis, especially the hepatoduodenal region, and the inclusion of the localization of the metastatic lymph nodes in the synoptic reports is beneficial for the patients in terms of better prognostic classification. Furthermore, the detection of lymph node metastasis in specific sites associated with adverse prognosis, such as the hepatoduodenal region, may affect the treatment protocol when detected postoperatively, or may indicate the candidates for neoadjuvant chemotherapy when detected preoperatively, and may be a topic for future studies.

## Conflict of Interest

There is no conflict of interest.
